# Gluten-Free Bread Enriched with Potato and Cricket Powder: Comparative Study of the Effects of Protein on Physicochemical Properties Bonds and Molecular Interactions

**DOI:** 10.3390/foods14111959

**Published:** 2025-05-30

**Authors:** Jakub Królak, Jan Jakub Kucharski, Przemysław Łukasz Kowalczewski, Klaudia Dudek, Millena Ruszkowska, Paweł Jeżowski, Łukasz Masewicz, Przemysław Siejak, Hanna Maria Baranowska

**Affiliations:** 1 Department of Food Technology of Plant Origin, Faculty of Food Science and Nutrition, Poznań University of Life Sciences, 60-624 Poznań, Poland; jakub.krolak@up.poznan.pl; 2 Students’ Scientific Club of Food Technologists, Faculty of Food Science and Nutrition, Poznań University of Life Sciences, 60-624 Poznań, Poland; jan.kucharskii@outlook.com (J.J.K.); klaudia.dudek.16.kd@gmail.com (K.D.); 3 Department of Quality Management, Faculty of Management and Quality Science, Gdynia Maritime University, 81-225 Gdynia, Poland; m.ruszkowska@wznj.umg.edu.pl; 4 Institute of Chemistry and Technical Electrochemistry, Faculty of Chemical Technology, Poznan University of Technology, 60-965 Poznań, Poland; pawel.jezowski@put.poznan.pl; 5 Department of Physics and Biophysics, Faculty of Food Science and Nutrition, Poznań University of Life Sciences, 60-637 Poznań, Poland; lukasz.masewicz@up.poznan.pl (Ł.M.); przemyslaw.siejak@up.poznan.pl (P.S.); hanna.baranowska@up.poznan.pl (H.M.B.)

**Keywords:** sustainable protein, insect-derived protein, potato-derived protein, circular economy

## Abstract

The increasing demand for diverse foods and tailored nutrition encourages the development of innovative products, such as bread enriched with cricket powder (CP) or potato protein (PP). This study presents the preparation and analysis of gluten-free breads with CP and PP, focusing on their nutritional value and physical properties. Analytical methods included water activity measurement, bread volume, crumb color analysis, FTIR spectroscopy, low-field NMR relaxometry, and texture profile analysis. Ash content ranged from 0.60 ± 0.03% to 1.16 ± 0.11%, and caloric values ranged from 216.2 to 229.5 kcal/100 g. Water activity remained stable across all samples (0.975–0.976). Crumb color analysis showed the greatest change in CP samples (ΔE = 14.07), while PP had minimal impact (ΔE = 2.15). FTIR spectra revealed increased amide I and II bands, indicating higher protein content. NMR results demonstrated shorter T_1_, T_21_, and T_22_ times for CP, suggesting reduced water mobility and a denser structure, while PP samples showed higher values, indicating a looser, more hydrated matrix. Texture analysis confirmed that CP increased firmness and compactness, whereas PP enhanced springiness. These findings suggest that CP and PP can improve the nutritional and structural properties of gluten-free bread, offering valuable alternatives for modern dietary needs.

## 1. Introduction

As our society grows and become larger, our requirements for more specific foods become more varied [1]. One notable example of the growing demand for gluten-free products is bread. This product can be the answer to some diseases, like non-celiac gluten sensitivity (NCGS), wheat allergy, or irritable bowel syndrome [2]. Celiac disease is an immune reaction triggered by the consumption of gluten found in wheat, rye, barley, and related grains in individuals with a genetic predisposition. In contrast, wheat allergy occurs when insoluble gliadins in wheat interact with immunoglobulin E (IgE), leading to allergic reactions that can be life-threatening. Unlike celiac disease, wheat allergy does not cause lasting damage to the gastrointestinal system [3]. Speaking of gluten-free bread, many consumers accustomed to traditional wheat or wheat–rye bread may find it quite unusual. This is due to its texture and taste. These breads tend to have a less flexible crumb that hardens quicker and crumbles more easily. Their taste is also usually less attractive to customers [4,5]. Comparing the nutritional values of wheat and gluten-free products, it can be seen that gluten-free products have more fat, less protein, and fewer minerals than wheat products [2,6]. Nowadays, we are looking for new sources of proteins and mineral compounds to enrich our diets. This movement goes hand in hand with the search for new food additives. Two examples of this are potato proteins and cricket powders. These two products can enhance the value of the gluten-free bread.

Potato protein (PP) can be obtained from potato wastewater, which is a byproduct of potato processing [7]. In recent years, there has been growing interest in the application of potato proteins, particularly those extracted from potato juice, as functional ingredients in gluten-free bread formulations. This interest stems from their potential to compensate for common deficiencies in gluten-free products, such as low protein content, poor crumb structure, and limited sensory appeal. Potato proteins are characterized by a high digestible indispensable amino acid score (DIAAS), a complete amino acid profile including branched-chain amino acids, and the presence of bioactive compounds such as phenolic acids, flavonoids, and anthocyanins, which contribute antioxidant and health-promoting properties [8,9,10]. Moreover, their functional attributes—such as emulsifying, foaming, and gelling capacities—along with the possibility of structural modification (e.g., through glycosylation), facilitate improvements in dough viscoelasticity, specific volume, and crumb texture [11,12]. Literature evidence confirms that the incorporation of moderate levels of potato proteins (typically 2–5%) positively affects the quality of gluten-free bread by enhancing its nutritional value, texture, and sensory characteristics [13,14]. Importantly, these proteins are derived from potato juice (a byproduct of starch production), positioning them as a sustainable alternative to conventional protein sources [15,16], in line with current trends in circular economy practices and the development of environmentally conscious functional foods. Utilizing potato wastewater through sustainable methods not only reduces waste but also transforms it into valuable resources for various industries, like the food industry [17,18,19].

The application of cricket (*Acheta domesticus*) powder (CP) in gluten-free bread production represents an innovative approach to enhancing both the nutritional value and bioactive potential of these products. Crickets are a highly nutritious and sustainable food source, rich in high-quality protein, essential amino acids, healthy fats, vitamins, and minerals such as iron, zinc, and B_12_ [20,21]. From a sensory and technological standpoint, cricket powder contributes to the development of desirable caramel and roasted flavor notes and influences textural parameters such as hardness and water dynamics, potentially affecting shelf-life and freshness [22]. Despite these numerous advantages, potential allergenicity and safety concerns, including microbiological contamination and heavy metal content, must be carefully managed [23]. Importantly, the use of cricket powder also aligns with sustainable food production goals due to its considerably lower environmental footprint compared to conventional livestock farming [24]. Its protein content often exceeds that of traditional livestock, making it an excellent alternative for addressing global protein demands. Additionally, crickets have a lower environmental footprint, requiring less land, water, and feed compared to conventional animal farming [25,26]. The high nutritional value of crickets, combined with their sustainability, makes them a promising option for future food security and dietary diversification. Incorporating crickets into human diets can help combat malnutrition while promoting eco-friendly food production [27].

Using these two ingredients can affect gluten-free bread, adding flavor and crunchiness and improving the outside and inside look, which could attract more people to try gluten-free products. Given the known shortcomings of gluten-free bread and the beneficial properties of potato and cricket proteins, the aim of the present study was to evaluate the impact of these protein additives on the nutritional value, as well as the physical and molecular properties of gluten-free bread.

## 2. Materials and Methods

### 2.1. Raw Materials

Corn and rice flours were purchased from Melvit S.A. (Warsaw, Poland), guar gum from Guangrao Liuhe Chemical Co., Ltd. (Qingdao, China), citrus pectin—Aglupectin LA-SX224—from Silvateam S.p.a. (San Michele di Mondovì, Italy), baker’s yeast from Lallemand Polska Sp. z o.o. (Józefow, Poland), refined sugar from Pfeifer & Langen Polska S.A. (Poznań, Poland), refined salt from CENOS Sp. z o.o. (Września, Poland), rapeseed oil from Bunge Poland (Kruszwica, Poland), and inulin—Orafti^®^HPX—from Beneo GmbH (Mannheim, Germany). Cricket powder was bought from Frutavita Sp. z o.o. (Kietrz, Poland). The potato protein concentrate (in powder form) used in this study was obtained from potato juice using our proprietary patented method, described in detail previously [9].

### 2.2. Dough Preparation and Baking

The bread dough was prepared using the single-phase method. The bread was prepared using a mixture of rice flour and corn flour in a 4:1 ratio, guar gum, citrus pectin, freeze-dried baker’s yeast, sugar, salt, rapeseed oil, cricket powder (denoted as CP), potato protein (denoted as PP), inulin, and water. Based on the manufacturer’s data, CP contains 68.3% protein, 19.6% fat, and 5.1% fiber. In the applied PP, the following composition was determined: 63.4% protein, 0.26% fat, and 7.54% mineral compounds. The temperature of the water for the preparation of the dough was chosen so that the resulting dough reached 35 °C. The detailed formulation of the analyzed bread is presented in Table 1.

All the ingredients without oil were mixed with a KitchenAid mixer (model 5KPM5EWH, KitchenAid Ariston, Benton Harbor, MI, USA) for 2 min. The oil was then added and mixed for a further 6 min. Next, the dough was removed from the mixing bowl, shaped into molds, and placed in the fermentation chamber at 37 °C and a relative humidity of 75% (RH) for 20 min. The bread was baked in a baker’s oven (MIWE Michael Wenz GmbH, Amstein, Germany) at 230 °C for 25 min. The bread was allowed to cool for 2 h at 20 °C, then weighed and analyzed.

### 2.3. Proximate Composition

Total nitrogen content was assessed using the Kjeldahl method, in compliance with ISO 20483 [28], and was subsequently used to determine the protein content (P) by applying a conversion factor of 5.7. The ash content was analyzed following the ISO 2171 [29] standard, while the total fat content (F) was measured according to AACC 30-25.01 [30]. Moisture content was evaluated based on the AACCI 44-19.01 [31] standard. The proximate carbohydrate content (C) was estimated by subtracting the total amounts of ash, fat, protein, and moisture from 100%. Additionally, the energy value (EV) was calculated using the following formula [32]:

EV (kcal/100 g) = 4 × (P + C) + 9 × F

### 2.4. Water Activity Measurement

Water activity (a_w_) was measured using an AquaLab 4TE instrument (AS42.14.0. Decagon Devices, Inc., Pullman, WA, USA) with an accuracy of ±0.0003 at 20.0 ± 2.5 °C.

### 2.5. Bread Quality Evaluation

Bread volume was evaluated following the AACC 10-05.01 standard procedure [33]. Additionally, baking loss and cooling loss were calculated according to the methods described by Leuschner et al. [34].

### 2.6. Bread Crumb Color Analysis

The crumb color was assessed utilizing a Chroma Meter CR-410 (Konica Minolta Sensing Inc., Tokyo, Japan). Color variations were documented on the CIE L*a*b* scale, where L* represents lightness, while a* and b* indicate redness and yellowness, respectively. Each sample underwent 10 individual measurements. Furthermore, the total color difference (∆E) was determined using the following equation [35]:

$$∆E=\sqrt{{∆L}^{2}+{∆a}^{2}+{∆b}^{2}}$$

Additionally, the color analysis included the calculation of the Whiteness Index (WI). The WI was determined using the following formula:

$$WI=\sqrt{100-{\left(100-L\right)}^{2}+a^{2}+b^{2}}$$

### 2.7. FTIR Analysis

FTIR spectra were recorded using a Perkin Elmer spectrophotometer (Waltham, MA, USA) fitted with an ATR accessory featuring a diamond internal reflection element. The measurements covered a spectral range of 4000 to 500 cm^−1^. Since some important peaks of proteins (especially in the Amide I and Amide II region) overlap with bands characteristic of O-H vibrations, originating from water present in the samples, the samples’ spectra were measured against the water spectrum as a reference to minimize the influence of water on the recorded results. This approach allows the expression of some peaks of lower intensity; however, it can result in negative values of spectra, especially in regions characteristic of O-H vibrations (stretching at 3500–2900 cm^−1^ and bending at 1700–1400 cm^−1^ regions).

### 2.8. Low-Field NMR Relaxometry

Crumb samples with a volume of 1.5 cm^3^ were placed in measurement tubes and securely sealed with Parafilm^®^. The determination of spin-lattice (T_1_) and spin-spin (T_2_) relaxation times was carried out using a pulse NMR spectrometer PS15T operating at 15 MHz (Ellab, Poznań, Poland). The measurements were performed at a controlled temperature of 21.0 ± 0.5 °C. For T_1_ relaxation time measurements, the inversion-recovery (180−t−90) [36] pulse sequence was employed. The interval between RF pulses (t) ranged from 20 to 80 ms, with a repetition time of 10 s. In each case, 32 FID signals were recorded, collecting 119 data points from each signal. The CracSpin software [37] was utilized to calculate spin-lattice relaxation times, applying the ‘spin grouping’ method for data analysis. The fitting of multiexponential decays was executed using Marquardt’s minimization algorithm. The accuracy of the relaxation parameters was assessed based on the standard deviation. The temporal evolution of the FID signal amplitude at the applied impulse frequency is expressed by the following equation:

$$M_{z}\left(t\right)=M_{0}\left\{1-2\exp\left(\frac{-t}{T_{1}}\right)\right\}$$

where M_z_(t) is the actual magnetization value; M_0_ is the equilibrium magnetization value.

A monoexponential recovery of magnetization was observed, indicating that the system relaxes according to a single T_1_ spin-lattice relaxation time.

The spin-spin (T_2_) relaxation times were determined using a pulse sequence based on the Carr-Purcell-Meiboom-Gill (CPMG) spin echo method (90 − t/2 − (180)_n_) [38,39]. The interval (t) between consecutive 180° RF pulses ranged from 0.5 to 0.8 ms, with a repetition time of 10 s. A total of 100 spin echoes (n) were recorded, and five signal accumulations were performed. To calculate the spin-spin relaxation time, the echo amplitudes were fitted to the appropriate equation [40]:

$$M_{x.y}\left(t\right)=M_{0}\sum_{i=1}^{n}p_{i}\exp\left[\frac{-t}{T_{2i}}\right]$$

where M_x.y_ (t) is the echo amplitude; M_0_ is the equilibrium amplitude; p_i_ is the fraction of protons relaxing with the T_2i_ spin–spin time.

### 2.9. Texture Analysis

The Texture Profile Analysis (TPA) of the bread was conducted one day after baking. A TA.XTplus texture analyzer (Stable Micro Systems Co., Ltd., Godalming, UK) with a 5 kg load cell was utilized for the measurements. The 15 samples underwent double compression using a cylindrical probe with a diameter of 35 mm. The testing parameters were configured as follows: pre-test speed—5.0 mm/s, test speed—5.0 mm/s, post-test speed—7.0 mm/s, and strain—40%. Bread loaves were sliced into 25 mm thick pieces (discarding the end slices) and analyzed or calculated for firmness, springiness, cohesiveness, chewiness, and resilience using Exponent Connect software (Stable Micro Systems Co., Ltd., Godalming, UK).

### 2.10. Statistical Analysis

The statistical analyses were performed using Statistica 13.3 (TIBCO Software Inc., Palo Alto, CA, USA). One-way analysis of variance and Tukey’s post hoc test was performed to determine statistically homogenous subsets at α = 0.05.

## 3. Results and Discussion

### 3.1. Nutritional Value of Analyzed Breads

The results of the proximate analysis of the gluten-free breads are presented in Table 2. The enrichment of gluten-free breads with CP and PP significantly enhanced their protein content. The reference sample (R), formulated without the addition of protein-rich ingredients, exhibited the lowest protein content. The incorporation of CP and PP resulted in a statistically significant increase (*p* < 0.05) in protein content across all enriched formulations. The highest protein concentration was observed in the PP100 sample, composed exclusively of PP. A gradual increase in protein content was evident with increasing proportions of PP in the CP/PP blend, confirming the high protein-contributing potential of this plant-based ingredient [7]. Notably, the use of CP alone (CP100) more than doubled the protein content compared to the R bread.

Ash content followed a decreasing trend with increasing PP concentration. The highest ash levels were recorded in samples containing higher amounts of CP (CP100-CP60PP40), which reflects the mineral richness of insect-based ingredients [21,41]. Conversely, lower ash values in the PP-dominant formulations, particularly PP100, are indicative of the comparatively lower mineral content of the plant-derived protein isolate [16]. The fat content in all bread samples remained within a narrow range and did not differ significantly (*p* > 0.05) among most formulations. The fat present in the analyzed samples comes mainly from fat added to the recipe. Despite the high fat content in CP [42], its amount was not significant enough to change the chemical composition of the bread portions. As expected, carbohydrate content decreased proportionally with the substitution of starch-based ingredients for protein-rich ones. The reference bread showed the highest carbohydrate content, whereas the lowest value was recorded for the PP100 sample. This trend directly corresponds to the reduction in flour mix content and its replacement with CP and PP. Despite variations in macronutrient composition, the energy values across all samples remained relatively consistent, ranging from 216.2 to 229.5 kcal/100 g. Interestingly, breads containing only cricket powder (CP100) were slightly less caloric than the reference sample, which can be attributed to their lower carbohydrate content and moderate levels of protein and fat.

Water activity (a_w_) values were comparable across all formulations (0.975–0.976), indicating that neither CP nor PP additions adversely affected the microbiological stability of the final products [43].

### 3.2. Bread Volume, Baking and Cooling Losses

The results regarding the volume of gluten-free breads and their baking losses are presented in Table 3. The R bread, which did not contain any protein enrichment, exhibited the highest loaf volume, regardless of having the highest baking loss. The addition of CP as the sole protein source (CP100) did not significantly affect loaf volume. However, it significantly (*p* < 0.05) reduced both direct baking loss and total baking loss. This effect may be attributed to the presence of fat and fiber in the CP, which likely contributed to improved water retention and enhanced crumb structure stability during baking [44,45].

A gradual replacement of CP with PP resulted in a consistent decrease in bread volume. These findings suggest that PP has a limited capacity to support gas retention and expansion in gluten-free doughs compared to cricket-derived protein, which may exhibit emulsifying and foam-stabilizing properties. Despite the decline in loaf volume, almost all protein-enriched formulations (CP and PP) were associated with significantly lower baking losses compared to the R bread, except for CP100. The lowest baking loss was seen for the PP100 sample, which may reflect the high water-binding capacity of PP [16] and its ability to reduce water evaporation during baking, as reported in another study [46]. The obtained results indicate that CP has a favorable impact on bread volume and structure, and thus an appropriate balance between both protein sources may lead to optimized bread characteristics. Cooling loss, defined as the moisture loss occurring after baking during the bread cooling phase, is a critical quality parameter influencing crumb moistness, shelf life, and textural properties. In the reference bread (R), the cooling loss was 2.58%, which can be considered a moderate value for gluten-free formulations. The replacement of part of the flour mixture with cricket powder (CP) alone (CP100) did not significantly alter this parameter (2.38%), suggesting that CP had a negligible impact on moisture migration during the cooling phase. However, the introduction of potato protein (PP), either alone or in combination with CP, noticeably affected the cooling loss. The sample containing 20% CP and 80% PP (CP20PP80) showed a slight reduction (2.11%), while the formulation with 40% CP and 60% PP (CP40PP60) exhibited the lowest cooling loss among all tested breads (1.85%). This suggests that PP at higher concentrations may enhance water retention post-baking, likely due to its superior water-binding and film-forming properties, which limit surface evaporation during cooling. In contrast, the CP80PP20 sample displayed the highest cooling loss (3.21%), significantly exceeding that of the reference. This anomaly may be explained by structural instability resulting from the predominance of cricket powder combined with a relatively low amount of PP, leading to a less cohesive crumb matrix more prone to post-baking dehydration. The elevated standard deviation for bread volume in this group also suggests variability in structure that could influence water retention. Interestingly, while increasing the PP content from 20% to 100% generally led to reduced cooling loss, the relationship was not strictly linear, indicating potential interactions between CP and PP affecting moisture dynamics. Overall, the results suggest that higher levels of PP improve moisture retention during cooling, while CP has a limited or formulation-dependent effect on this parameter.

### 3.3. Crumb Color Analysis

**Figure 1:**
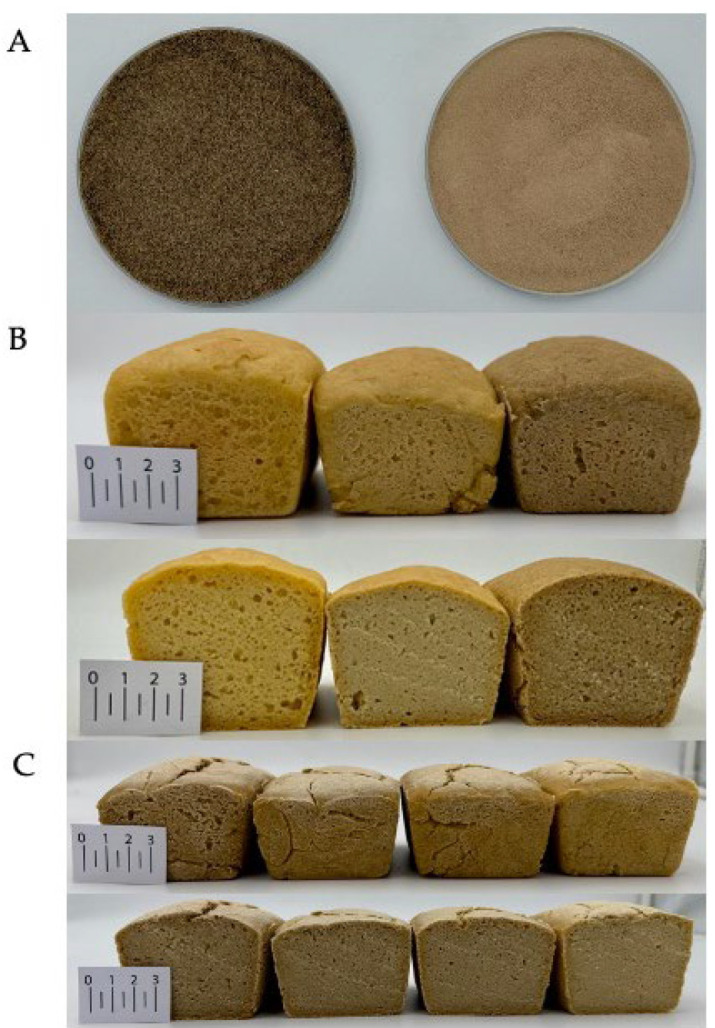
(**A**) Representative images of the protein sources used in the study (left CP, right PP); (**B**) visual comparison of the extreme formulation variants (R, PP100, and CP100, respectively), including whole loaves and their cross-sections; (**C**) comparison of the remaining bread variants containing different proportions of cricket powder (CP) and plant protein (PP), including whole loaves and their cross-sections (from left: CP80PP20, CP60PP40, CP40PP60, CP20PP80).

The crumb color varied significantly depending on the type and proportion of the protein ingredient used (Table 4). The lightness parameter (L*) was highest in the reference sample (R), while the darkest crumb was observed in the bread containing only cricket powder (CP100). Partial replacement of CP with PP gradually increased crumb lightness. Further substitution with PP led to a continuous increase in lightness, reaching 68.26 in the PP100, closely approximating the reference bread. This indicates that PP has little impact on darkening the crumb, contrary to CP, which significantly reduces lightness. The obtained results are consistent with the literature data describing the effect of PP on the color of wheat bread [46] and CP on the color of gluten-free bread [47]. A similar trend was observed for the a* parameter, which reflects the red–green axis. The CP100 sample exhibited the highest redness, which decreased progressively as the proportion of CP decreased. The b* values, representing the yellow–blue axis, were highest in the reference bread, while the samples with added proteins showed values without a clear trend linked to the protein source. The total color difference (ΔE), calculated in relation to the reference sample, confirmed the above observations. The most noticeable color change was observed in CP100 (ΔE > 14), whereas gradual substitution of CP with PP resulted in a decrease in ΔE, down to 2.15 in PP100. A color difference of less than 3 is imperceptible to a consumer inexperienced in assessing color [35]. Figure 1 presents photographs illustrating (A) the protein sources; (B) a comparison of the extreme variants (i.e., R, PP100, and CP100); and (C) a comparison of the remaining formulations with varying proportions of CP and PP, including whole loaves and their cross-sections. The whiteness index (WI) also showed considerable variation across the samples—the lowest WI was found in CP100 (55.07), while the highest was recorded in the reference sample (67.35). Breads enriched with PP had higher WI values than those containing higher proportions of CP. The color changes observed align with the anticipated trend, given that CP exhibits markedly lower brightness than PP; therefore, the incorporation of increasing proportions of CP resulted in a progressively darker appearance of the bread. CP significantly influenced the crumb color, leading to darker shades and enhanced red tones, likely due to the presence of melanin and Maillard reaction products formed during roasting insects [48,49]. In contrast, PP contributed to a lighter crumb appearance, making it a valuable functional ingredient for improving the sensory acceptability of gluten-free breads [50,51].

### 3.4. Fourier Transform Infrared Spectroscopy

**Figure 2:**
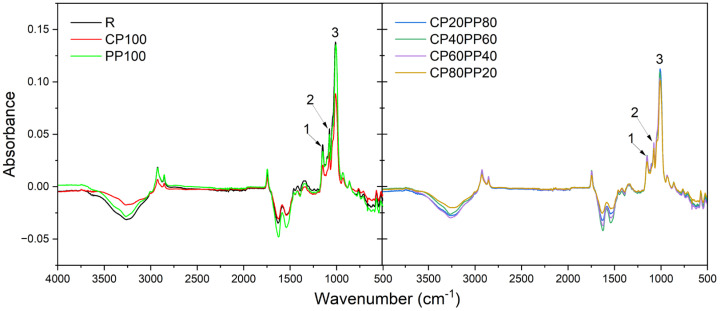
Fourier-transform infrared spectroscopy spectra of analyzed breads. 1–3: peak numbers (described in Table 5).

The FTIR spectral analysis (Figure 2) enabled the assessment of the impact of CP and PP enrichment on the chemical profile of bread crumbs. The recorded spectra in the range of 4000–500 cm^−1^ reflected the presence of major functional groups typical for proteins, carbohydrates, and lipids. The region between 3300 and 3000 cm^−1^ showed bands associated with O-H and N-H stretching vibrations, indicating the presence of both water molecules and peptides (peptide A and peptide B). Bands around 2920 cm^−1^, attributed to C-H stretching vibrations (CH_2_ and CH_3_), also remained relatively stable across all formulations, in agreement with the comparable fat content (Table 2). The most pronounced changes were observed in the 1600–1500 cm^−1^ region, where the amide I and II bands became increasingly intense in samples with higher CP and PP content (e.g., CP100, PP100). The direct assessment of protein content based on those bands is difficult due to the presence of water in the samples. Since bands characteristic of -OH bonds overlap each protein-indicating band, the influence of water and the presence and state of molecules (bonding interactions, leading to different mobility of water molecules within the matrix, as shown by the NMR experiment), makes direct assessment of protein content based on FTIR spectra difficult. However, Figure 1 shows that there is no clear relationship between protein content and the intensity of the amide bands (for both regions: amide A, B, and amide I, II), indicating strong interactions between peptides and water molecules. This supports the finding on different water interactions (bonding) with the matrix. The region between 1150 and 950 cm^−1^, mainly associated with carbohydrate-related vibrations (C-O and C-C), is not influenced by water molecules, allowing conclusions to be drawn about the carbohydrate content in the samples. Those bands showed a gradual decrease in intensity with increasing protein enrichment (Figure 2 and Table 5), consistent with the reduction in carbohydrate content. The negative values in the FTIR spectra (regions of 3500–2900 cm^−1^ and 1700–1400 cm^−1^) result from lower water content in samples compared to the reference (water).

### 3.5. LF NMR Relaxometry

The LF NMR analysis revealed substantial differences in water mobility within the crumb matrix of gluten-free breads enriched with CP and PP (Table 6). The spin-lattice relaxation time (T_1_), representing the mobility of free water [52,53], was highest in the reference sample (R = 122.29 ms), consistent with its low protein content (11.15%) and high carbohydrate fraction (44.27%), which are typical for conventional gluten-free breads. The addition of CP notably reduced T_1_ values, with the lowest observed in CP80PP20 (80.42 ms), indicating a pronounced restriction of water mobility. This suggests that CP strongly binds water molecules [54,55,56]. A progressive increase in PP content partially mitigated this effect, resulting in slightly higher T_1_ values in CP20PP80 (85.04 ms) and PP100 (85.05 ms), which remained below the reference, confirming enhanced water structuring in protein-enriched systems.

The transverse relaxation component T_21_, corresponding to tightly bound water [52,53], followed a similar trend. The CP100 sample exhibited the shortest T_21_ (4.32 ms), suggesting strong water–protein interactions [57,58], likely attributable to the specific amino acid profile and matrix compaction provided by insect proteins. In contrast, increasing proportions of PP resulted in longer T_21_ values (up to 9.12 ms in PP100), implying a comparatively looser water-binding network. The T_22_ component, assigned to moderately bound water (e.g., within protein-starch gels) [36,59], was drastically reduced in CP100 (33.62 ms) compared to R (69.56 ms), supporting the hypothesis of a denser, more aggregated matrix induced by CP. With increasing PP levels, T_22_ values increased, reaching levels nearly identical to the reference in CP20PP80 (67.62 ms) and PP100 (69.95 ms), which may be attributed to the gel-forming capacity of PP and its role in maintaining matrix hydration.

Despite these structural shifts in water mobility, the water activity (a_w_) remained statistically unchanged (Table 1) across all samples, indicating that the differences observed in relaxation behavior result from changes in the physical state of water rather than its total content. The obtained NMR results indicate that CP introduction significantly modifies the crumb microstructure by enhancing water immobilization, while PP counterbalances this effect, improving water distribution and potentially contributing to more favorable textural and shelf-life properties.

### 3.6. Textural Properties

The textural properties of gluten-free breads enriched with CP and PP, presented in Table 7, were closely linked to the changes in water mobility observed through LF NMR analysis. Firmness increased significantly with higher CP content, with CP100 showing the highest value (258 N), consistent with its denser structure and stronger water binding, as indicated by the reduced T_1_ and T_21_ values in the NMR results (Table 6). In contrast, the springiness decreased as CP content increased, with CP100 showing the lowest springiness (88%), reflecting the restricted water mobility and reduced elasticity of the crumb matrix.

The cohesiveness and chewiness also increased with higher CP content (Table 6), with CP100 exhibiting the highest values (0.49 and 1446, respectively), indicating a more compact and chewy texture due to the tight binding of water molecules. Protein blends with higher PP content, such as CP40PP60 and CP20PP80, displayed lower firmness values but higher springiness (92–94%), indicating that PP helped maintain a more elastic structure. This is further supported by the NMR data, where PP-enriched samples had higher T_22_ values, suggesting a more flexible water distribution. Resilience, which measures the ability to recover after compression, was highest in the PP100 sample (0.32), likely due to the gel-forming properties of PP that enhanced the bread’s ability to spring back. The results show that CP contributes to a denser, chewier texture, while PP enhances elasticity, resilience, and water distribution, thus optimizing both the structural and sensory properties of gluten-free breads.

## 4. Application Potential and Research Limitations

The motivation for this study was to support the development of nutritionally enhanced gluten-free bread formulations, particularly for individuals with celiac disease, non-celiac gluten sensitivity, or wheat allergy. These consumers often face limited options that are both safe and nutritionally adequate. The significant increase in protein content observed in all enriched samples directly addresses a key deficiency of typical gluten-free products. Moreover, the improved texture, reduced baking loss, and favorable water-binding properties demonstrated in this study indicate that such formulations may offer improved shelf-life. Therefore, the developed products have the potential to provide both technological and nutritional benefits for gluten-intolerant individuals.

Despite the promising results, several limitations of the present study should be acknowledged. The primary focus was placed on evaluating the effect of potato and cricket protein enrichment on the general nutritional value and technological properties of gluten-free bread. However, while protein content is an important indicator, it does not fully reflect the nutritional quality of the product. Key aspects such as amino acid composition, protein digestibility, and bioavailability were not assessed in this study, yet they are critical for understanding the real nutritional contribution of the enriched breads. Furthermore, both potato protein and cricket powder contain not only proteins but also a variety of other nutrients and bioactive compounds—including dietary fiber, phenolics, flavonoids, and chitin—that may affect the nutritional value, bioactivity, and potentially even the health-promoting properties of the final products. In addition, these components, along with the changes in texture and color, may influence consumer acceptance and sensory appeal. Future research should aim to comprehensively evaluate the amino acid profile, digestibility, and potential health benefits of the developed formulations, as well as to conduct consumer studies and in vitro or in vivo assessments of bioactivity.

## 5. Conclusions

This study demonstrates the potential of PP and CP as valuable ingredients for enhancing the nutritional and overall qualities of gluten-free bread. The incorporation of these proteins significantly improved the protein content, enriching gluten-free bread significantly and improving their nutritional profile without compromising energy content. FTIR spectral analysis confirmed that CP and PP enrichment altered the chemical composition of gluten-free bread by enhancing protein-associated bands and reducing carbohydrate-related signals. Low-field NMR relaxometry revealed that the addition of CP and PP positively influenced water binding and distribution within the crumb matrix; however, synergistic effects of both protein sources were observed at the molecular level. The findings suggest that PP and CP can effectively address the common deficiencies in gluten-free products, such as low protein content. Moreover, the use of these sustainable protein sources aligns with circular economy practices and promotes food security. Future research should focus on optimizing the proportions of PP and CP to achieve the best balance between nutritional value and sensory properties, as well as exploring their potential applications in other gluten-free products. This research is particularly important for people with celiac disease, as it offers a nutritionally enhanced gluten-free bread option that addresses common deficiencies in protein.

## Figures and Tables

**Table 1:** Bread composition details.

Ingredient (%)	R	CP100	CP80PP20	CP60PP40	CP40PP60	CP20PP80	PP100
Flour mix	50.00	46.67	46.67	46.67	46.67	46.67	46.67
Guar gum	1.00	1.00	1.00	1.00	1.00	1.00	1.00
Pectin	1.00	1.00	1.00	1.00	1.00	1.00	1.00
Baker’s yeast	2.20	2.20	2.20	2.20	2.20	2.20	2.20
Sugar	0.90	0.90	0.90	0.90	0.90	0.90	0.90
Salt	0.80	0.80	0.80	0.80	0.80	0.80	0.80
Rapeseed oil	1.60	1.60	1.60	1.60	1.60	1.60	1.60
Potato protein	0.00	0.00	0.67	1.33	2.00	2.67	3.33
Cricket powder	0.00	3.33	2.67	2.00	1.33	0.67	0.00
Inulin	0.83	0.83	0.83	0.83	0.83	0.83	0.83
Water	41.67	41.67	41.67	41.67	41.67	41.67	41.67

**Table 2:** Proximate composition, energy value, and water activity (a_w_) of breads.

Sample	Protein Content (%)	Ash Content (%)	Fat Content (%)	Carbohydrate Content (%) ^1^	Energy Value (kcal/100 g) ^2^	a_w_ (-)
R	11.15 ± 0.89 ^c^	0.60 ± 0.03 ^c^	0.87 ± 0.02 ^b^	44.27	229.5	0.976 ± 0.001 ^a^
CP100	23.05 ± 1.43 ^b^	1.16 ± 0.11 ^a^	0.92 ± 0.03 ^a^	29.05	216.7	0.975 ± 0.001 ^a^
CP80PP20	23.53 ± 1.06 ^b^	1.12 ± 0.06 ^a^	0.90 ± 0.03 ^a^	28.70	217.0	0.975 ± 0.001 ^a^
CP60PP40	25.72 ± 0.97 ^ab^	1.01 ± 0.10 ^a^	0.89 ± 0.05 ^a^	26.69	217.6	0.975 ± 0.001 ^a^
CP40PP60	28.46 ± 1.88 ^a^	0.92 ± 0.04 ^ab^	0.87 ± 0.04 ^ab^	25.41	216.2	0.975 ± 0.001 ^a^
CP20PP80	29.99 ± 1.21 ^a^	0.82 ± 0.07 ^b^	0.83 ± 0.02 ^b^	22.60	217.8	0.975 ± 0.001 ^a^
PP100	30.73 ± 2.23 ^a^	0.78 ± 0.09 ^b^	0.83 ± 0.04 ^b^	21.97	218.2	0.976 ± 0.001 ^a^

Values marked with the same lowercase letter in columns do not differ significantly, *p* > 0.05. ^1^ The carbohydrate content was estimated by subtracting the average content of ash, fat, moisture, and protein from 100%. ^2^ Energy value was calculated based on the average moisture, protein, fat, and carbohydrate content.

**Table 3:** Volume of bread leaves, baking and cooling losses.

Sample	Bread Volume (mL/100 g)	Baking Loss (%)	Cooling Loss (%)
R	800 ± 18 ^a^	15.47 ± 3.55 ^a^	2.58 ± 0.11 ^b^
CP100	800 ± 10 ^a^	10.50 ± 3.01 ^ab^	2.38 ± 0.09 ^b^
CP80PP20	733 ± 58 ^ab^	10.69 ± 1.73 ^b^	3.21 ± 0.11 ^a^
CP60PP40	600 ± 11 ^b^	9.27 ± 1.61 ^b^	2.78 ± 0.09 ^b^
CP40PP60	570 ± 47 ^b^	9.80 ± 1.82 ^b^	1.85 ± 0.16 ^c^
CP20PP80	567 ± 26 ^b^	9.63 ± 0.93 ^b^	2.11 ± 0.11 ^bc^
PP100	516 ± 38 ^b^	9.03 ± 1.05 ^b^	2.35 ± 0.06 ^b^

Values marked with the same lowercase letter in columns do not differ significantly, *p* > 0.05.

**Table 4:** Color parameters of analyzed breads.

Sample	L*	a*	b*	ΔE	WI
Protein source					
CP	47.12 ± 0.40	3.76 ± 0.03	9.64 ± 0.13	-	-
PP	63.03 ± 0.77	4.75 ± 0.07	12.53 ± 0.52	-	-
Breads					
R	70.40 ± 1.00 ^a^	−1.01 ± 0.06 ^e^	13.75 ± 0.34 ^a^	-	67.35
CP100	56.34 ± 0.12 ^d^	2.70 ± 0.02 ^a^	10.26 ± 0.06 ^b^	14.07	55.07
CP80PP20	58.05 ± 0.45 ^c^	2.39 ± 0.06 ^b^	10.51 ± 0.06 ^b^	13.21	56.70
CP60PP40	58.31 ± 0.74 ^c^	2.21 ± 0.09 ^b^	9.97 ± 0.18 ^b^	13.07	57.08
CP40PP60	62.58 ± 0.29 ^b^	1.55 ± 0.02 ^c^	10.36 ± 0.04 ^b^	8.90	61.14
CP20PP80	63.52 ± 0.13 ^b^	1.16 ± 0.13 ^c^	10.62 ± 0.04 ^b^	7.86	61.98
PP100	68.26 ± 0.82 ^a^	0.44 ± 0.05 ^d^	10.27 ± 0.13 ^b^	2.15	66.63

Values marked with the same lowercase letter in columns do not differ significantly, *p* > 0.05. ΔE—total color difference, WI—whiteness index.

**Table 5:** Intensities of FTIR bands assigned to carbohydrates.

Peak Number	Wavenumber (cm^−1^)	Absorbance Value of the Sample						
		R	CP100	PP100	CP80PP20	CP60PP40	CP40PP60	CP20PP80
1	1150	0.0336	0.0298	0.0238	0.0292	0.0401	0.0206	0.0284
2	1078	0.0498	0.0415	0.0363	0.0419	0.0552	0.0325	0.0417
3	1014–1008	0.1355	0.1093	0.1013	0.1033	0.1380	0.0887	0.1123

**Table 6:** Results of ^1^H NMR study for bread.

Sample	T_1_ (ms)	T_21_ (ms)	T_22_ (ms)
R	122.3 ± 0.8 ^a^	10.71 ± 0.21 ^a^	69.56 ± 0.76 ^a^
CP100	99.1 ± 0.8 ^b^	4.32 ± 0.14 ^d^	33.62 ± 0.88 ^d^
CP80PP20	80.4 ± 0.7 ^e^	9.42 ± 0.19 ^b^	69.92 ± 0.71 ^a^
CP60PP40	101.7 ± 0.8 ^b^	8.72 ± 0.20 ^c^	53.14 ± 0.69 ^b^
CP40PP60	88.3 ± 0.6 ^c^	8.68 ± 0.43 ^c^	49.14 ± 0.83 ^c^
CP20PP80	85.0 ± 0.9 ^d^	9.08 ± 0.32 ^bc^	67.62 ± 0.68 ^a^
PP100	85.0 ± 0.6 ^d^	9.12 ± 0.27 ^b^	69.95 ± 0.77 ^a^

Values marked with the same lowercase letter in columns do not differ significantly, *p* > 0.05.

**Table 7:** Textural properties of breadcrumbs.

Sample	Firmness (N)	Springiness (%)	Cohesiveness (-)	Chewiness (-)	Resilience (-)
R	122 ± 24 ^c^	97 ± 1 ^a^	0.44 ± 0.05 ^b^	531 ± 197 ^c^	0.20 ± 0.05 ^ab^
CP100	258 ± 13 ^a^	88 ± 4 ^b^	0.49 ± 0.05 ^ab^	1446 ± 43 ^a^	0.25 ± 0.03 ^b^
CP80PP20	213 ± 35 ^a^	93 ± 1 ^ab^	0.50 ± 0.03 ^ab^	1343 ± 245 ^a^	0.25 ± 0.02 ^ab^
CP60PP40	194 ± 30 ^a^	89 ± 5 ^b^	0.52 ± 0.05 ^ab^	1333 ± 167 ^a^	0.27 ± 0.02 ^ab^
CP40PP60	159 ± 27 ^b^	92 ± 2 ^ab^	0.55 ± 0.02 ^a^	1258 ± 132 ^a^	0.29 ± 0.02 ^ab^
CP20PP80	148 ± 31 ^b^	94 ± 2 ^ab^	0.57 ± 0.02 ^a^	1047 ± 111 ^ab^	0.30 ± 0.01 ^a^
PP100	144 ± 14 ^b^	93 ± 1 ^ab^	0.58 ± 0.03 ^a^	912 ± 199 ^b^	0.32 ± 0.04 ^a^

Values marked with the same lowercase letter in columns do not differ significantly, *p* > 0.05.

## Data Availability

The datasets generated for this study are available upon request to the corresponding author.

## References

[1] Gu D., Andreev K., Dupre M.E. (2021). Major Trends in Population Growth Around the World. China CDC Wkly..

[2] Rybicka I. (2018). The Handbook of Minerals on a Gluten-Free Diet. Nutrients.

[3] Skendi A., Papageorgiou M., Varzakas T. (2021). High Protein Substitutes for Gluten in Gluten-Free Bread. Foods.

[4] do Nascimento A.B., Fiates G.M.R., dos Anjos A., Teixeira E. (2014). Gluten-free is not enough—Perception and suggestions of celiac consumers. Int. J. Food Sci. Nutr..

[5] Roman L., Belorio M., Gomez M. (2019). Gluten-Free Breads: The Gap Between Research and Commercial Reality. Compr. Rev. Food Sci. Food Saf..

[6] Šmídová Z., Rysová J. (2022). Gluten-Free Bread and Bakery Products Technology. Foods.

[7] Kowalczewski P.Ł., Olejnik A., Świtek S., Bzducha-Wróbel A., Kubiak P., Kujawska M., Lewandowicz G. (2022). Bioactive compounds of potato (*Solanum tuberosum* L.) juice: From industry waste to food and medical applications. CRC Crit. Rev. Plant Sci..

[8] Herreman L.C.M., de Vos A.M., Cosijn M.M., Tjalma L.F., Spelbrink R.E.J., van der Voort Maarschalk K., Laus M.C. (2024). Potato: A Sustainable Source of Functional and Nutritional Proteins. Sustainable Protein Sources.

[9] Kowalczewski P.Ł., Olejnik A., Białas W., Rybicka I., Zielińska-Dawidziak M., Siger A., Kubiak P., Lewandowicz G. (2019). The Nutritional Value and Biological Activity of Concentrated Protein Fraction of Potato Juice. Nutrients.

[10] Pęksa A., Miedzianka J. (2021). Potato Industry By-Products as a Source of Protein with Beneficial Nutritional, Functional, Health-Promoting and Antimicrobial Properties. Appl. Sci..

[11] Liu X., Ji Y., Wang H., Zhang Y., Zhang H. (2023). Effect of glycosylated potato protein on the characteristics of gluten-free dough and steamed bread. Int. J. Food Sci. Technol..

[12] Akbari N., Mohammadzadeh Milani J., Biparva P. (2020). Functional and conformational properties of proteolytic enzyme-modified potato protein isolate. J. Sci. Food Agric..

[13] Witczak T., Juszczak L., Ziobro R., Korus J. (2017). Rheology of gluten-free dough and physical characteristics of bread with potato protein. J. Food Process Eng..

[14] Liu X., Mu T., Sun H., Zhang M., Chen J., Fauconnier M.L. (2019). Effect of ingredients on the quality of gluten-free steamed bread based on potato flour. J. Food Sci. Technol..

[15] Alting A.C., Pouvreau L., Giuseppin M.L.F., van Nieuwenhuijzen N.H. (2011). Potato proteins. Handbook of Food Proteins.

[16] Jeżowski P., Polcyn K., Tomkowiak A., Rybicka I., Radzikowska D. (2020). Technological and antioxidant properties of proteins obtained from waste potato juice. Open Life Sci..

[17] Kot A.M., Pobiega K., Piwowarek K., Kieliszek M., Błażejak S., Gniewosz M., Lipińska E. (2020). Biotechnological Methods of Management and Utilization of Potato Industry Waste—A Review. Potato Res..

[18] Bzducha-Wróbel A., Koczoń P., Błażejak S., Kozera J., Kieliszek M. (2019). Valorization of Deproteinated Potato Juice Water into β-Glucan Preparation of C. utilis Origin: Comparative Study of Preparations Obtained by Two Isolation Methods. Waste Biomass Valoriz..

[19] Bzducha-Wróbel A., Pobiega K., Błażejak S., Kieliszek M. (2018). The scale-up cultivation of Candida utilis in waste potato juice water with glycerol affects biomass and β(1,3)/(1,6)-glucan characteristic and yield. Appl. Microbiol. Biotechnol..

[20] van Huis A. (2013). Potential of insects as food and feed in assuring food security. Annu. Rev. Entomol..

[21] Zielińska E., Baraniak B., Karaś M., Rybczyńska K., Jakubczyk A. (2015). Selected species of edible insects as a source of nutrient composition. Food Res. Int..

[22] Wieczorek M., Kowalczewski P., Drabińska N., Różańska M., Jeleń H. (2022). Effect of Cricket Powder Incorporation on the Profile of Volatile Organic Compounds, Free Amino Acids and Sensory Properties of Gluten-Free Bread. Polish J. Food Nutr. Sci..

[23] Hassan S.A., Altemimi A.B., Hashmi A.A., Shahzadi S., Mujahid W., Ali A., Bhat Z.F., Naz S., Nawaz A., Abdi G. (2024). Edible crickets as a possible way to curb protein-energy malnutrition: Nutritional status, food applications, and safety concerns. Food Chem. X.

[24] da Rosa Machado C., Thys R.C.S. (2019). Cricket powder (*Gryllus assimilis*) as a new alternative protein source for gluten-free breads. Innov. Food Sci. Emerg. Technol..

[25] Fu C., Cheema W.A., Mobashar M., Shah A.A., Alqahtani M.M. (2025). Insects as Sustainable Feed: Enhancing Animal Nutrition and Reducing Livestock Environmental Impression. J. Anim. Physiol. Anim. Nutr..

[26] Lisboa H.M., Nascimento A., Arruda A., Sarinho A., Lima J., Batista L., Dantas M.F., Andrade R. (2024). Unlocking the Potential of Insect-Based Proteins: Sustainable Solutions for Global Food Security and Nutrition. Foods.

[27] Magara H.J.O., Niassy S., Ayieko M.A., Mukundamago M., Egonyu J.P., Tanga C.M., Kimathi E.K., Ongere J.O., Fiaboe K.K.M., Hugel S. (2021). Edible Crickets (Orthoptera) Around the World: Distribution, Nutritional Value, and Other Benefits—A Review. Front. Nutr..

[28] (2013). Cereals and pulses—Determination of the Nitrogen Content and Calculation of the Crude Protein Content—Kjeldahl Method.

[29] (2007). Cereals, Pulses and by-Products—Determination of Ash Yield by Incineration.

[30] AACC (2000). Crude fat in wheat, corn, and soy flour, feeds, and mixed feeds. AACC Int. Approv. Methods.

[31] AACC (2009). 44-19.01 Moisture—Air-oven method, drying at 135 degrees. AACC International Approved Methods.

[32] Montowska M., Kowalczewski P.Ł., Rybicka I., Fornal E. (2019). Nutritional value, protein and peptide composition of edible cricket powders. Food Chem..

[33] AACCI (2009). 10-05.01 Guidelines for Measurement of Volume by Rapeseed Displacement. AACC International Approved Methods.

[34] Leuschner R.G.K., O’Callaghan M.J.A., Arendt E.K. (1997). Optimization of baking parameters of part-baked and rebaked Irish brown soda bread by evaluation of some quality characteristics. Int. J. Food Sci. Technol..

[35] Mokrzycki W., Tatol M. (2011). Color difference Delta E—A survey. Mach. Graph. Vis..

[36] Brosio E., Gianferri R.R., Brosio E. (2009). Low-resolution NMR—An analytical tool in foods characterization and traceability. Basic NMR in Foods Characterization.

[37] Węglarz W.P., Harańczyk H. (2000). Two-dimensional analysis of the nuclear relaxation function in the time domain: The program CracSpin. J. Phys. D. Appl. Phys..

[38] Carr H.Y., Purcell E.M. (1954). Effects of Diffusion on Free Precession in Nuclear Magnetic Resonance Experiments. Phys. Rev..

[39] Meiboom S., Gill D. (1958). Modified Spin-Echo Method for Measuring Nuclear Relaxation Times. Rev. Sci. Instrum..

[40] Baranowska H.M. (2011). Water molecular properties in forcemeats and finely ground sausages containing plant fat. Food Biophys..

[41] Ayieko M.A., Ogola H.J., Ayieko I.A. (2016). Introducing rearing crickets (gryllids) at household levels: Adoption, processing and nutritional values. J. Insects Food Feed.

[42] Ho I., Peterson A., Madden J., Huang E., Amin S., Lammert A. (2022). Will It Cricket? Product Development and Evaluation of Cricket (*Acheta domesticus*) Powder Replacement in Sausage, Pasta, and Brownies. Foods.

[43] Tapia M.S., Alzamora S.M., Chirife J., Barbosa-Cánovas G.V., Fontana A.J., Schmidt S.J., Labuza T.P. (2020). Effects of Water Activity (aw) on Microbial Stability as a Hurdle in Food Preservation. Water Activity in Foods.

[44] Bresciani A., Cardone G., Jucker C., Savoldelli S., Marti A. (2022). Technological Performance of Cricket Powder (*Acheta domesticus* L.) in Wheat-Based Formulations. Insects.

[45] Bawa M., Songsermpong S., Kaewtapee C., Chanput W. (2020). Nutritional, sensory, and texture quality of bread and cookie enriched with house cricket (*Acheta domesticus*) powder. J. Food Process. Preserv..

[46] Kowalczewski P., Różańska M., Makowska A., Jeżowski P., Kubiak P. (2019). Production of wheat bread with spray-dried potato juice: Influence on dough and bread characteristics. Food Sci. Technol. Int..

[47] Kowalczewski P.Ł., Gumienna M., Rybicka I., Górna B., Sarbak P., Dziedzic K., Kmiecik D. (2021). Nutritional Value and Biological Activity of Gluten-Free Bread Enriched with Cricket Powder. Molecules.

[48] Nath P., Pandey N., Samota M., Sharma K., Kale S., Kannaujia P., Sethi S., Chauhan O.P. (2022). Browning Reactions in Foods. Advances in Food Chemistry.

[49] Starowicz M., Zieliński H. (2019). How Maillard Reaction Influences Sensorial Properties (Color, Flavor and Texture) of Food Products?. Food Rev. Int..

[50] Guiné R.P.F. (2022). Textural Properties of Bakery Products: A Review of Instrumental and Sensory Evaluation Studies. Appl. Sci..

[51] Monteiro J.S., Farage P., Zandonadi R.P., Botelho R.B.A., de Oliveira L.L., Raposo A., Shakeel F., Alshehri S., Mahdi W.A., Araújo W.M.C. (2021). A Systematic Review on Gluten-Free Bread Formulations Using Specific Volume as a Quality Indicator. Foods.

[52] Kuhn W. (1990). NMR Microscopy—Fundamentals, Limits and Possible Applications. Angew. Chem. Int. Ed. Engl..

[53] Kirtil E., Oztop M.H. (2016). 1H Nuclear Magnetic Resonance Relaxometry and Magnetic Resonance Imaging and Applications in Food Science and Processing. Food Eng. Rev..

[54] Hirsch A.J. (2018). Functional Properties of Protein and Chitin from Commercial Cricket Flour. Master’s Thesis.

[55] Ruggeri M., Bianchi E., Vigani B., Sánchez-Espejo R., Spano M., Totaro Fila C., Mannina L., Viseras C., Rossi S., Sandri G. (2023). Nutritional and Functional Properties of Novel Italian Spray-Dried Cricket Powder. Antioxidants.

[56] Kowalczewski P.Ł., Siejak P., Jarzębski M., Jakubowicz J., Jeżowski P., Walkowiak K., Smarzyński K., Ostrowska-Ligęza E., Baranowska H.M. (2023). Comparison of technological and physicochemical properties of cricket powders of different origin. J. Insects Food Feed.

[57] McDonnell C.K., Allen P., Morin C., Lyng J.G. (2014). The effect of ultrasonic salting on protein and water–protein interactions in meat. Food Chem..

[58] Cônsolo N.R.B., de Paula A.P.M., Rezende-de-Souza J.H., Herreira V.L.S., Laura S.M., Gôngora A., Colnago L.A., Moraes T.B., Santos P.M., Nassu R.T. (2024). Assessment of water relaxometry of meat under different ageing processes using time domain nuclear magnetic resonance relaxometry. Food Res. Int..

[59] Kazemi-Taskooh Z., Varidi M. (2023). How can plant-based protein–polysaccharide interactions affect the properties of binary hydrogels? (A review). Food Funct..

